# Cell Type-Specific Modulation of Cobalamin Uptake by Bovine Serum

**DOI:** 10.1371/journal.pone.0167044

**Published:** 2016-11-28

**Authors:** Hua Zhao, Kalani Ruberu, Hongyun Li, Brett Garner

**Affiliations:** 1 Illawarra Health and Medical Research Institute, University of Wollongong, Wollongong, NSW, Australia; 2 School of Biological Sciences, University of Wollongong, Wollongong, NSW, Australia; Bauer Research foundation, UNITED STATES

## Abstract

Tracking cellular ^57^Co-labelled cobalamin (^57^Co-Cbl) uptake is a well-established method for studying Cbl homeostasis. Previous studies established that bovine serum is not generally permissive for cellular Cbl uptake when used as a supplement in cell culture medium, whereas supplementation with human serum promotes cellular Cbl uptake. The underlying reasons for these differences are not fully defined. In the current study we address this question. We extend earlier observations by showing that fetal calf serum inhibits cellular ^57^Co-Cbl uptake by HT1080 cells (a fibrosarcoma-derived fibroblast cell line). Furthermore, we discovered that a simple heat-treatment protocol (95°C for 10 min) ameliorates this inhibitory activity for HT1080 cell ^57^Co-Cbl uptake. We provide evidence that the very high level of haptocorrin in bovine serum (as compared to human serum) is responsible for this inhibitory activity. We suggest that bovine haptocorrin competes with cell-derived transcobalamin for Cbl binding, and that cellular Cbl uptake may be minimised in the presence of large amounts of bovine haptocorrin that are present under routine *in vitro* cell culture conditions. In experiments conducted with AG01518 cells (a neonatal foreskin-derived fibroblast cell line), overall cellular ^57^Co-Cbl uptake was 86% lower than for HT1080 cells, cellular TC production was below levels detectable by western blotting, and heat treatment of fetal calf serum resulted in only a modest increase in cellular ^57^Co-Cbl uptake. We recommend a careful assessment of cell culture protocols should be conducted in order to determine the potential benefits that heat-treated bovine serum may provide for *in vitro* studies of mammalian cell lines.

## Introduction

Cobalamin (Cbl), which is also commonly referred to as vitamin B12, is required for erythrocyte formation, DNA synthesis, and the maintenance of neurological function [1–4]. As described in detail previously [2, 5], methyl Cbl (MeCbl) and adenosyl Cbl (AdoCbl) are the forms of B12 that are active in human metabolism. Several Cbl deficiency states exist in humans, some of which are caused by a loss of function in proteins that transport Cbl either to cells or within subcellular compartments [3, 6, 7]. For example, as discussed in detail previously [8], mutations in the several genes including those encoding TC (*TCN2*), HC (*TCN1*) and TCblR (*CD320*), result in a disruption in cellular Cbl uptake and a concomitant cellular and tissue Cbl deficiency [9–12].

A substantial body of research spanning more than four decades has relied on *in vitro* cell culture studies as an import approach to defining the precise pathways involved in the intra- and extra-cellular transport of Cbl and the molecular defects that may occur in genetic causes of Cbl deficiency. As described in detail previously [2, 5], the key proteins involved in extracellular Cbl transport in humans are transcobalamin (TC), intrinsic factor (IF) and haptocorrin (HC) [3, 13, 14]. Dietary Cbl initially binds to HC in saliva before being released to IF in the duodenum. After intestinal absorption, the IF-Cbl complex is transported through the intracellular lysosomal compartment and subsequently secreted by multidrug resistance protein 1 (MRP1/ABCC1) [15], into the portal circulation before transport to peripheral cells as a TC-Cbl complex [16]. The TC-Cbl is then endocytosed by the transcobalamin receptor (TCblR/CD320), which is present on most cells in human tissues [17]. Plasma HC also binds Cbl where it may play a role as a Cbl store, based on the fact that it is not taken up into cells by the TCblR. HC also plays a role in the clearance of other corrinoid Cbl analogues from the circulation. HC-Cbl (and HC bound corrinoids) are thought to be taken up by the liver asialoglycoprotein receptor [3]. Another specialised route for TC-Cbl uptake is via megalin expressed in the kidney [18]. This facilitates TC-Cbl reabsorption, delivering filtered TC-Cbl back to the bloodstream. Both megalin and cubilin are expressed in other cell types, including mammary cells, where these receptors may also play a specialised role in Cbl homeostasis [19, 20]. Although HC does not share the same high degree of specificity for Cbl binding as TC, HC has greater affinity for Cbl than TC [3].

In the modelling of cellular Cbl homeostasis *in vitro*, early studies indicated that cell culture growth medium containing FCS is not permissive for Cbl uptake [21–24]. This was thought to be due to a lack of TC as demonstrated by gel filtration of serum proteins that bind ^57^Co-Cbl [24]. To overcome this problem, protocols were developed to culture cells in the presence of FCS, then with ^57^Co-Cbl bound to human TC (purified from human serum, HS), or with HS as a source of TC [21–23, 25].

In the present studies we have reinvestigated this phenomenon in order to better understand the factors that make FCS non-permissive for cellular ^57^Co-Cbl uptake. In contrast to our expectation that this was due to low TC levels in FCS, our data indicate that the high HC content of bovine serum also prevents efficient cellular ^57^Co-Cbl uptake *in vitro*, and that this appears to be a cell type-specific phenomenon.

## Materials and Methods

### Cell culture

Experiments were performed using the human fibrosarcoma fibroblast cell cline (HT1080, ATCC #CCL-121) that was obtained from the American Type Culture Collection (ATCC, Manassas, VA, USA), and the AG01518 human foreskin fibroblast cell line that was obtained from the Coriell Cell Repository Coriell Institute, Camden, NJ, USA). Both HT1080 and AG01518 fibroblasts were cultured in Dulbecco's modified eagle medium (DMEM, Life Technologies, USA, Cat #12800–017), supplemented with 10% (v/v) FCS (Interpath, USA, Cat #SFBS), 100 μg/ml penicillin/streptomycin (Sigma, USA, Cat #P4333), and 2 mM glutamine (Invitrogen, USA, Cat #15140122) at 37°C in a humidified atmosphere containing 5% CO_2_. The methods for cellular uptake of ^57^Co-Cbl have been previously described in detail [5]. In brief, HT1080 cells and AG01518 cells were grown in 6-well plates in triplicate until they reached approximately 70% confluence, unless stated otherwise. Note that the doubling times for HT1080 cells and AG01518 cells are reported to be 20–24 h and 3 to 4 days, respectively [26, 27], and initial seeding densities were adjusted to ensure similar confluence at the point of ^57^Co-Cbl addition to the growth medium. The cells were metabolically labeled with ^57^Co-Cbl (0.025 μCi/ml, MP Biomedicals, USA, Cat. # 06B-430002) in DMEM with 10% (v/v) FCS or 10% (v/v) HS (Sigma, USA, Cat. # H4522) for up to 72 h at 37°C. As described in detail previously [28], the radioactive tracer molecule [^57^Co]cyanoCbl was provided by the supplier in batches of 10.5 μCi in a volume of 1 ml H_2_O containing 0.9% (v/v) benzyl alcohol. On the reference date provided by the manufacturer, 0.1 ml from each batch of [^57^Co]cyanoCbl yielded 2.0 x 10^6^ cpm. After evaporation to dryness and reconstitution in cell culture medium, the [^57^Co]cyanoCbl radioactivity was measured in a 0.1 ml aliquot to confirm radioactivity levels before use in experiments. For all experiments, the [^57^Co]cyanoCbl tracer was used within 2 months of the reference date. As an approximation, based on a specific activity for [^57^Co]cyanoCbl of 300 μCi / μg, the amount of [^57^Co]cyanoCbl used in experiments was ~ 8.3 x 10^−2^ ng/ml (at the time of use in experiments 1000 cpm equates to ~ 2.4 pg of [^57^Co]Cbl).

After incubation, the growth medium was collected, while the cells were then rinsed with phosphate buffered saline (PBS) and harvested with 1% (w/v) trypsin in pure DMEM without serum. The amount of ^57^Co-Cbl in each cell pellet and growth medium sample (subsequent to 5 min centrifugation at 600 x *g* to remove cell debris) was measured using a Wallace Gamma Counter (PerkinElmer, Finland). Where indicated adult bovine serum (Sigma, USA, Cat #B9433) was also used as a comparator for FCS during ^57^Co-Cbl uptake experiments.

For serum dose-dependence experiments, the concentrations of FCS or HS were diluted to 2%, 4%, 6%, 8% and 10% (v/v) in DMEM as indicated. For the time-course experiments, the incubation periods were routinely 2 h, 4 h, 8 h, 12 h, 24 h, and 48 h, unless stated otherwise. For serum heat treatment experiments, FCS and HS were heated at 95°C for 10 min (or at 100°C for 20 min or at 56°C for 30 min where indicated). The serum was then added into the DMEM and incubated with the cells. For Cbl binding experiments, FCS and HS were serially diluted with pure DMEM at 1:10, 1:25, 1:50, 1:100, 1:1,000, and 1:10,000 dilutions. The ^57^Co-Cbl was then added to the samples and incubated at 37°C for 1 h. The samples were then transferred to 30 kDa MW cut-off Amicon Ultra-15 Centrifugal Filter Units (Millipore, USA, Cat.# UFC903024) and centrifuged at 12,000 x *g* for 20 min. The filters were turned upside down and centrifuged for 2 min and the retentate solution that contained protein bound ^57^Co-Cbl (i.e. TC-Cbl ~44 kDa and HC-Cbl ~64 kDa) complex was measured for radioactivity as above. Similarly, free ^57^Co-Cbl (~1.3 kDa) was collected in the filtrate and radioactivity measured as above. For the experiments using anti-TC antibody or anti-HC antibody to investigate the mechanism of ^57^Co-Cbl uptake, either anti-TC mouse monoclonal antibody (1:100, Santa Cruz, USA, Cat. # Sc-137017) or anti-HC polyclonal antibody (1:100, Abcam, UK, Cat. # Ab118386) was added to growth medium containing 10% HS or 10% FCS or 10% heated FCS and incubated with the cells for 48 h.

Note that in all cell culture experiments, we have used ^57^Co-cyano Cbl as a radioactive tracer. Even though this form of Cbl is what we added to the cell culture medium, once the cyano Cbl enters the intracellular compartment, it is metabolized to methyl Cbl and adenosyl Cbl (as discussed in detail above). It is also possible that during the course of our studies (up to 72 h), a small amount of ^57^Co-Cbl is transported from the cell back to the medium in a modified form (different to the originally added ^57^Co-cyano Cbl). Since we have not analyzed the different chemical forms of Cbl in our experiments, we simply refer to the tracer as ^57^Co-Cbl.

### Western blotting

Cell lysates and cell culture supernatants were assessed by western blotting as described previously [5, 29]. Cell pellets were homogenised in lysis buffer (50mM Tris, 150mM NaCl, 0.1% (w/v) SDS, 0.5% (w/v) sodium deoxycholate, 1% (v/v) Triton X 100) buffer containing a Complete protease inhibitor cocktail (P8340, Sigma), prepared according to the manufacturers instructions. All samples from cell pellets and growth medium (containing ~15 to 30 μg protein assessed using the bicinchoninic acid (BCA) method [30]) were heated at 95°C for 10min with loading dye containing β-mercaptoethanol before being separated on 12% SDS-PAGE gels using a Mini-Protean II system (Bio-Rad, USA) at 150 V for 70 min and then transferred at 100 V for 30 min onto 0.45 μm nitrocellulose membranes (Bio-Rad, USA, Cat. # 162–0115) using a Mini-Trans-Blot Electrophoretic Transfer cell (Bio-Rad, USA). The membranes were blocked in 5% (w/v) non-fat milk powder in PBS for 1 h at 22°C and then probed with an anti-HC mouse monoclonal antibody (1:250, Abcam, UK, Cat. # Ab118386), an anti-TC polyclonal antibody (1:1,000, Santa Cruz, USA, Cat. # Sc-137017), and an anti-β-actin rabbit polyclonal antibody (1:10,000 Sigma, USA, Cat. # A5060) for 16 h at 4°C. The membranes were then incubated with the respective horseradish-peroxidase (HRP)-conjugated goat anti-mouse (1:4,000, Dako, Australia, Cat. # P0447) and goat anti-rabbit (1:4,000, Dako, Australia, Cat. # P0448) IgG antibodies for 1 h at 22°C. The blots were rinsed in PBS, and the proteins were detected using enhanced chemiluminescence (Amersham Biosciences, USA, Cat. # 28-9068-37). The membranes were exposed to ECL hyperfilm (Amersham Biosciences, USA), which was developed and scanned to produce representative images.

## Results

**Fig 1:**
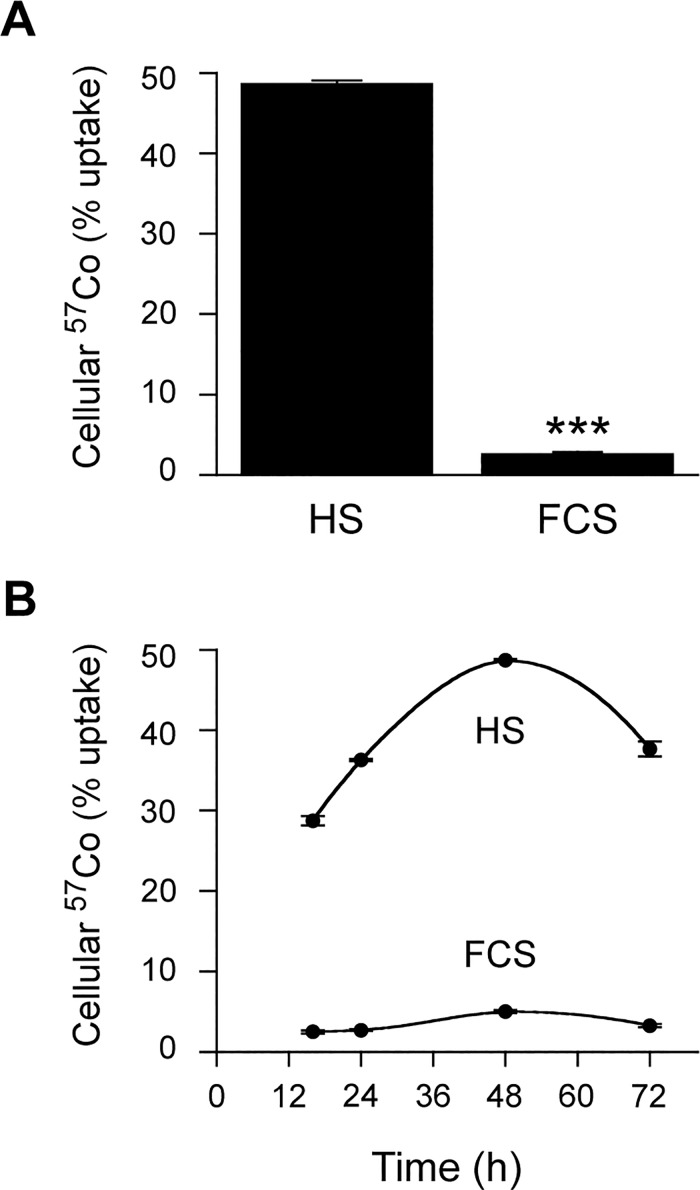
HT1080 cellular uptake of ^57^Co-Cbl in the presence of either HS or FCS. HT1080 cells were incubated at 37°C with ^57^Co-Cbl in DMEM containing either 10% HS or 10% FCS. ***A***, The cells were harvested for ^57^Co analysis after 48 h. ***B***, Under the same conditions used in “A”, a time-course study with samples taken at 16 h, 24 h, 48 h and 72 h was undertaken. Data are mean values + SE (n = 3).

We first examined the cellular uptake of ^57^Co-Cbl when HT1080 cells were cultured for up to 72 h in growth medium containing either 10% FCS or 10% HS. In both cases, the ^57^Co-Cbl was pre-incubated with serum (1 h at 37°C) to allow binding to serum proteins. In general agreement with earlier studies [21], we found that FCS was unable to support a high level of ^57^Co-Cbl uptake (Fig 1). When assessed at the 48 h time point, the amount of ^57^Co-Cbl detected in cells grown in FCS was 5.5% of the level detected in the cells grown in HS (Fig 1A). The overall amount of ^57^Co-Cbl present in the cells grown in HS increased with time up to 48 h, then the levels dropped at the 72 h time point as the cells formed a completely confluent monolayer. We found that the proportion of ^57^Co-Cbl detected in the cells grown in HS (expressed as a percentage of the total radioactivity in the cells and growth medium) varied between experiments from ~25 to ~50% (36.0% + 11.5%, mean + SE, n = 21 experiments).

**Fig 2:**
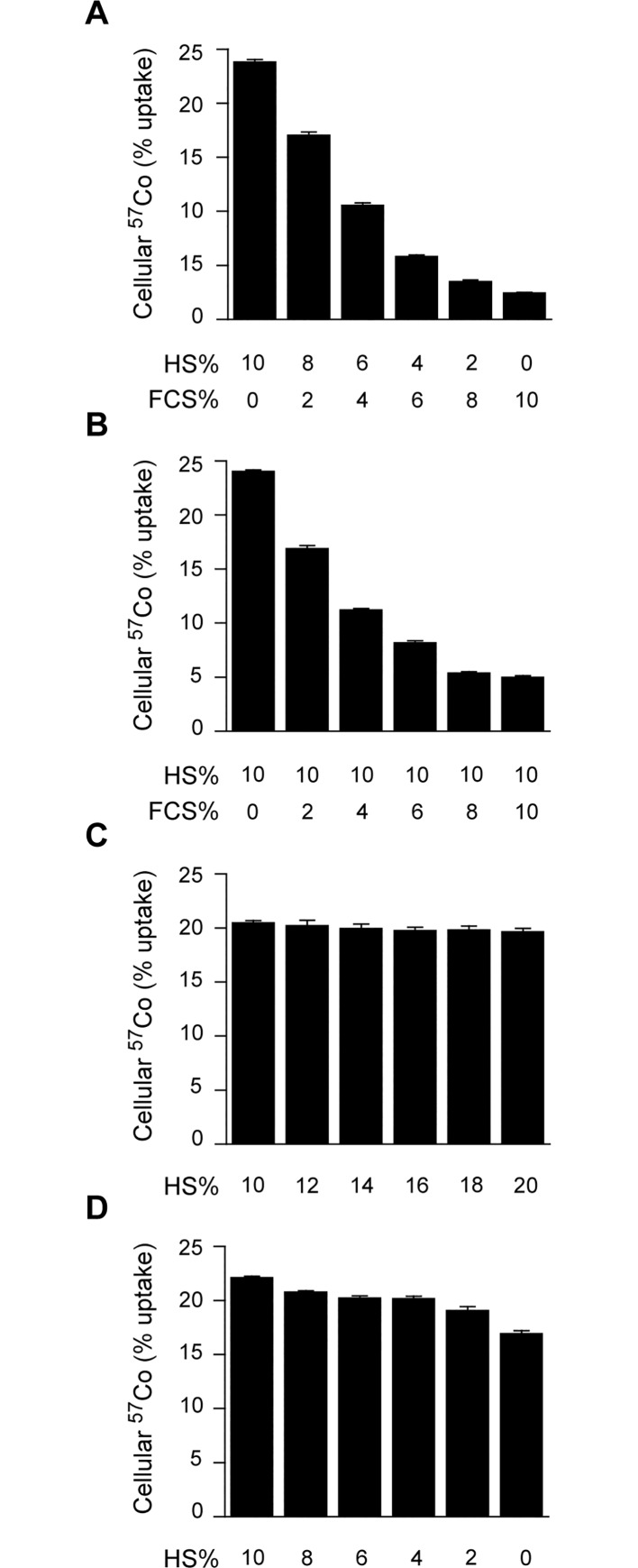
HT1080 cellular uptake of ^57^Co-Cbl in the presence of various concentrations of HS or FCS. HT1080 cells were incubated at 37°C with ^57^Co-Cbl in DMEM containing either HS or the given HS / FCS mixture. ***A*—*D***, In all experiments, the cells were harvested for ^57^Co analysis after 48 h. Data are mean values + SE (n = 3).

We reasoned that if the lack of cellular ^57^Co-Cbl uptake from FCS was due to the previously reported low levels of TC in FCS [24], that we might be able to use a mixture of HS and FCS that was sufficient to promote uptake based on the HS TC content. Keeping total growth medium serum levels at 10%, we found that even a minor adjustment in the serum composition (i.e. moving from 10% HS to 8% HS / 2% FCS) resulted in a significant 29% decrease (p < 0.0001) in ^57^Co-Cbl uptake (Fig 2A). This could suggest that the capacity for HS to bind ^57^Co-Cbl was close to saturation under our experimental conditions, or that FCS contains an inhibitory factor that prevents ^57^Co-Cbl uptake. To address the latter possibility, we used mixtures of serum that contain 10% HS as a constant, with additional increments of FCS. The addition of FCS dose-dependently inhibited cellular ^57^Co-Cbl uptake from the growth medium containing 10% HS (Fig 2B). Additional control experiments showed that increasing total HS contents incrementally above 10% had no impact on ^57^Co-Cbl uptake (Fig 2C). In addition, simple reduction of HS concentration in the medium only moderately reduced cellular ^57^Co-Cbl uptake (Fig 2D). For example, reducing the growth medium HS concentration by 80% (to 2% HS in total) reduced cellular ^57^Co-Cbl uptake by only 14% (p < 0.001). In the complete absence of HS (i.e. DMEM alone), cellular ^57^Co-Cbl uptake remained at a relatively high level (77% compared with cells cultured in 10% HS, Fig 2D). These experiments raised two questions related firstly to the nature of the factor in FCS that potently inhibits ^57^Co-Cbl uptake, and secondly to the mechanism by which HT1080 cells take up ^57^Co-Cbl in the apparent absence of a TC source.

**Fig 3:**
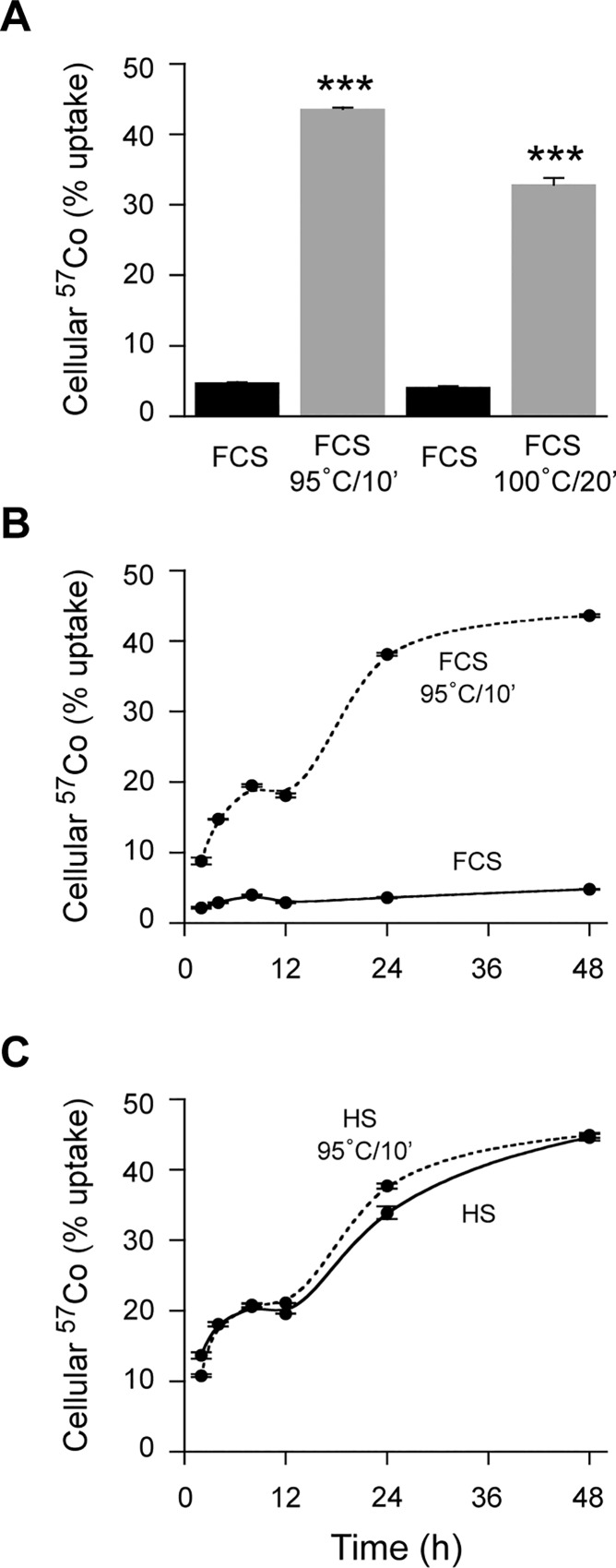
HT1080 cellular uptake of ^57^Co-Cbl in the presence of either FCS or HS with or without heat treatment. ***A*,** FCS was heated at either 95°C for 10 min or 100°C for 20 min before ^57^Co-Cbl addition, then incubated with HT1080 cells for 48 h and compared to standard FCS culture conditions. ***B*,** Using the same 95°C for 10 min FCS heating conditions used in “A”, a time-course study with samples taken at 2 h, 4 h, 8 h, 12 h, 24 h, and 48 h was undertaken. ***C***, For the purpose of comparison, a time-course study with heated HS (95°C for 10 min) was also conducted in parallel. Data are mean values + SE (n = 3).

To test the possibility that a heat-labile component of FCS (such as a protein) contributes to the inhibition of cellular ^57^Co-Cbl uptake we observed (Figs 1 and 2), we applied heat denaturation protocols to the FCS (either 95°C for 10 min or 100°C for 20 min) before the routine incubation of the serum with ^57^Co-Cbl to allow binding to native Cbl-binding proteins. These heat denaturation parameters were chosen based on standard protocols for protein denaturation, for example as used in PAGE, that are typically 95°C to 100°C for 5 to 10 min [31]. We found that heat denaturation essentially reversed the inhibitory capacity for FCS to block HT1080 cellular ^57^Co-Cbl uptake at the 48 h time point (Fig 3A). A more detailed time course experiment using heat-treated FCS (95°C for 10 min) revealed a biphasic kinetic for ^57^Co-Cbl uptake, with an initial rapid rate of uptake detected in the 2 h to 8 h time frame, that was followed by an apparent plateau, then a second sustained phase of uptake at the 24 h and 48 h time points (Fig 3B). This biphasic kinetic was almost identical in the HT1080 cells grown with HS (Fig 3C). Importantly, heat treatment of HS did not significantly increase cellular ^57^Co-Cbl uptake compared to the standard HS culture conditions (Fig 3C). In addition, when we heated serum at 56°C for 30 min (a protocol that is widely used to “heat-inactivate” serum in order to inactivate complement), this did not increase cellular ^57^Co-Cbl uptake compared to the standard culture conditions using either FCS or HS (S1 Fig).

Taken together, these experiments indicated that a heat-labile component of FCS was responsible for the inhibition of HT1080 cellular ^57^Co-Cbl uptake we observed, and that the mechanism of cellular ^57^Co-Cbl uptake may be independent of a serum-derived Cbl binding protein (i.e. TC).

**Fig 4:**
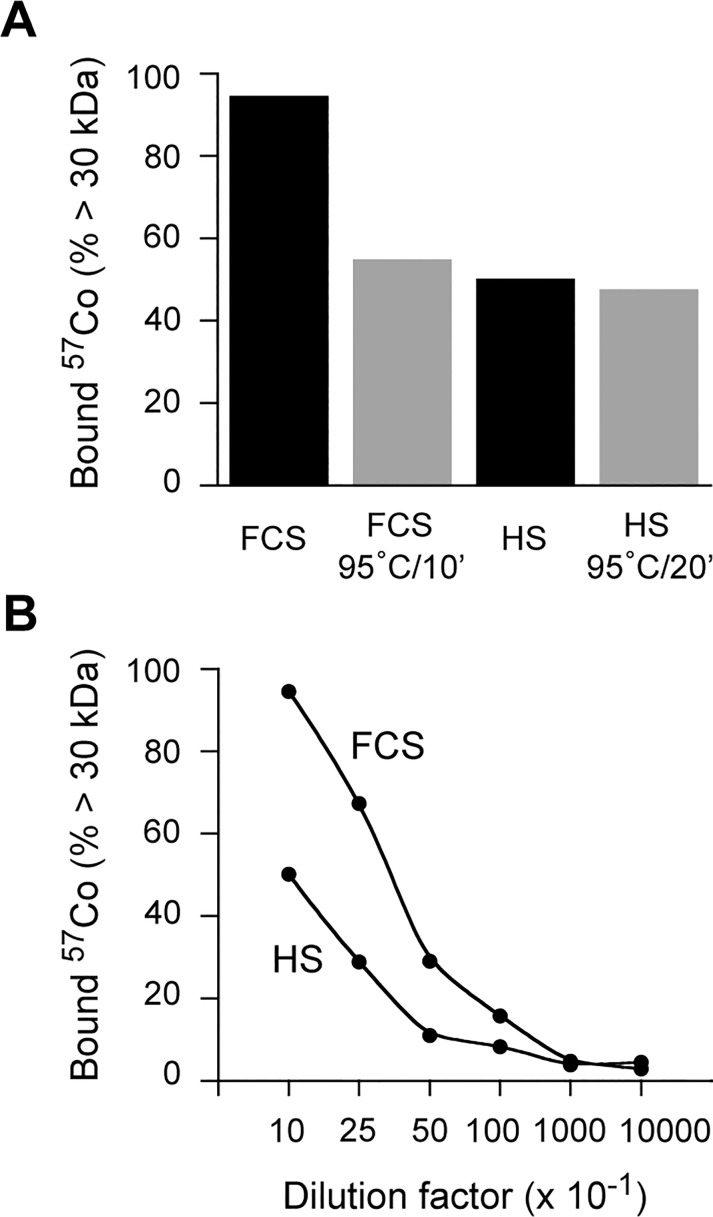
^57^Co-Cbl binding capacity of FCS or HS with or without heat treatment. FCS or HS was either not heated or heated at 95°C for 10 min before dilution to 10% in DMEM and subsequent incubation with ^57^Co-Cbl for 1 h at 37°C. The samples were then centrifuged with a centrifugal filtration device with a 30 kDa MW cut-off to assess the proportion of radioactivity bound to proteins (including HC and TC). ***A*,** The radioactivity of the retentate is expressed as a percentage of total radioactivity in each sample. FCS or HS were diluted with DMEM at 1:10, 1:25, 1:50, 1:100, 1:1,000, and 1:10,000 dilutions and subsequently incubation with ^57^Co-Cbl for 1 h at 37°C. ***B***, The samples were then centrifuged with a centrifugal filtration device as in “A”, and the proportion of radioactivity bound to proteins > 30 kDa assessed.

To assess for possible differences in the ability of FCS versus HS to bind ^57^Co-Cbl under our *in vitro* experimental conditions, we used a centrifugal filtration device with a 30 kDa MW cut-off to assess the proportion of radioactivity bound to proteins such as haptocorrin (HC) and TC (molecular weights of ~64 kDa and ~44 kDa, respectively). In these experiments, the serum was first diluted into DMEM in the range of 1/10 to 1/10,000 before ^57^Co-Cbl was added (see Materials and Methods section for further details). The data indicate that when diluted 1/10 in DMEM, both HS and FCS efficiently bind ^57^Co-Cbl (Fig 4A). There was a clear difference in the binding capacity, with FCS showing approximately double the binding capacity of HS, a difference that remained evident throughout the series of dilutions. Note that at the dilutions of 1/1000 and 1/10000, this difference between HS and FCS was no longer observed; however, this was at a point where only ~ 4% of the ^57^Co-Cbl remained in the >30 kDa fraction, a level that was very similar to the DMEM-only control (not shown), where 3% of the ^57^Co-Cbl radioactivity was recovered in the >30 kDa fraction. We conclude that both HS and FCS when diluted more than 1/1000 are unable to bind significant amounts of the added ^57^Co-Cbl, and that compared to HS, FCS has a superior binding capacity for ^57^Co-Cbl when diluted in DMEM.

We also assessed the capacity for heat-treated FCS and HS to bind ^57^Co-Cbl when diluted 1/10 in DMEM and found that heating (95°C for 10 min) reduced the ^57^Co-Cbl binding to FCS by 42.3% (i.e. the amount of ^57^Co-Cbl recovered in the > 30 kDa fraction was reduced from 95.0% to 54.8% with heating), whereas the same heat treatment of HS reduced ^57^Co-Cbl binding by only 6.5% (i.e. the amount of ^57^Co-Cbl recovered in the > 30 kDa fraction was reduced from 50.9% to 47.6% with heating). The fact that heat-treated diluted serum still binds ^57^Co-Cbl suggests low affinity / non-specific binding occurs or that a fraction of ^57^Co-Cbl-binding proteins (e.g. TC) may be extraordinarily heat-stable.

**Fig 5:**
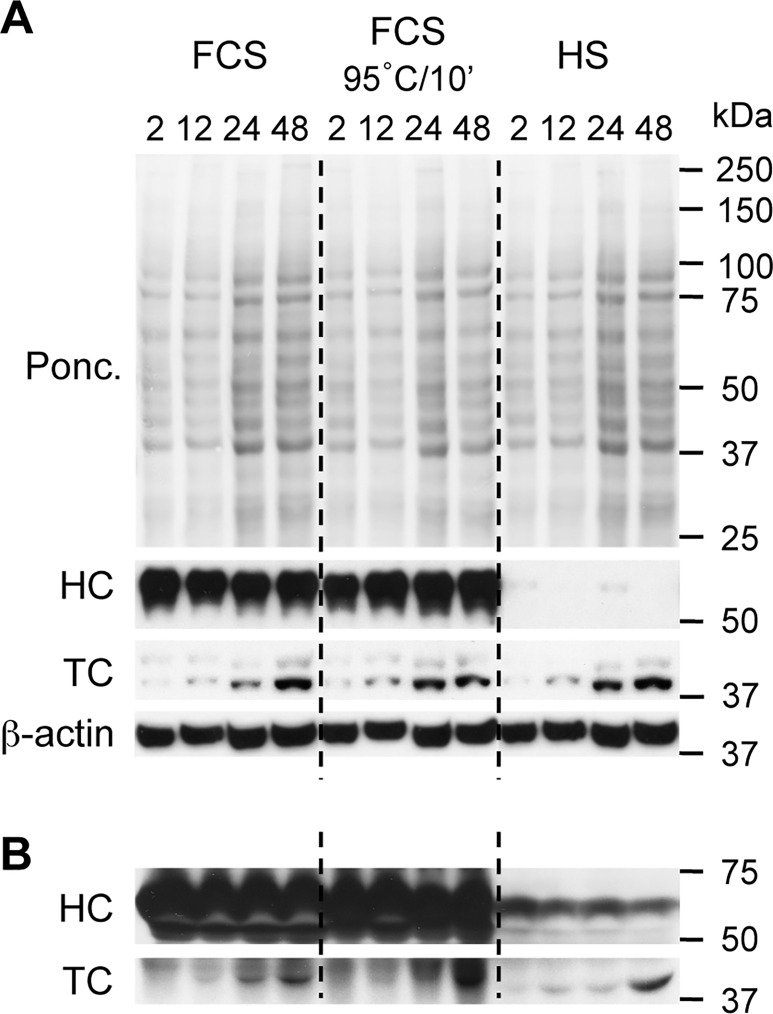
Assessment of haptocorrin and transcobalamin expression in HT1080 cells and growth medium. ***A***, HT1080 cells were cultured in DMEM containing either 10% FCS, 10% heat-treated (95°C for 10 min) FCS, or 10% HS, for 2 h, 12 h, 24 h and 48 h and the cells, and ***B***, cell culture medium supernatants, were collected and analysed for haptocorrin (HC), transcobalamin (TC), and β-actin by western blotting. Positions of molecular weight markers (kDa) are given on the right side of the blots. Ponc., Ponceau red stained membrane.

In order to assess the levels of both HC and TC in the HT1080 cells and culture medium under conditions that are both permissive and non-permissive for ^57^Co-Cbl uptake, we collected cell lysates and cell culture supernatants over the course of a 48 h experiment. We detected a strong signal for HC in the cell lysates grown in the FCS or heated FCS (Fig 5A) that did not change with cell growth (i.e. increases in total cell protein and β-actin were observed over time but cell-associated HC did not change). In contrast, only traces of a signal for HC were detected in cells grown in HS (Fig 5A). We concluded that the cell associated HC was likely derived from FCS and that FCS may therefore be enriched with HC as compared to HS. Western blotting of the cell culture supernatants confirmed this, as very high levels of HC were detected in the FCS-containing medium whereas in comparison, relatively low levels of HC were detected in the HS-containing medium (Fig 5B). It is noteworthy that the HC levels present in the HS-containing medium diminished over time, possibly indicating the total HC may have included residual bovine HC traces that were remaining after the initial routine culture of the cells in FCS.

In addition to the presence of HC, we also detected a clear signal for cellular TC under all HT1080 cell culture conditions. Importantly, cellular TC levels were at only trace levels at the 2 h and 12 h time points, with sharp increases in TC observed by the 24 h and 48 h time points (Fig 5A). The overall time course for TC appearance, as well as the levels detected, were very similar for all HT1080 culture conditions (Fig 5A). In addition, a similar time-dependent increase in TC detected in the HT1080 cell culture supernatants was also observed in all culture conditions (Fig 5B). The sharp increase in TC levels at the 24 h and 48 h time points coincides with the time-course for increased cellular ^57^Co-Cbl uptake we observed for HT1080 cells grown in either HS or heat-treated FCS (Fig 3).

**Fig 6:**
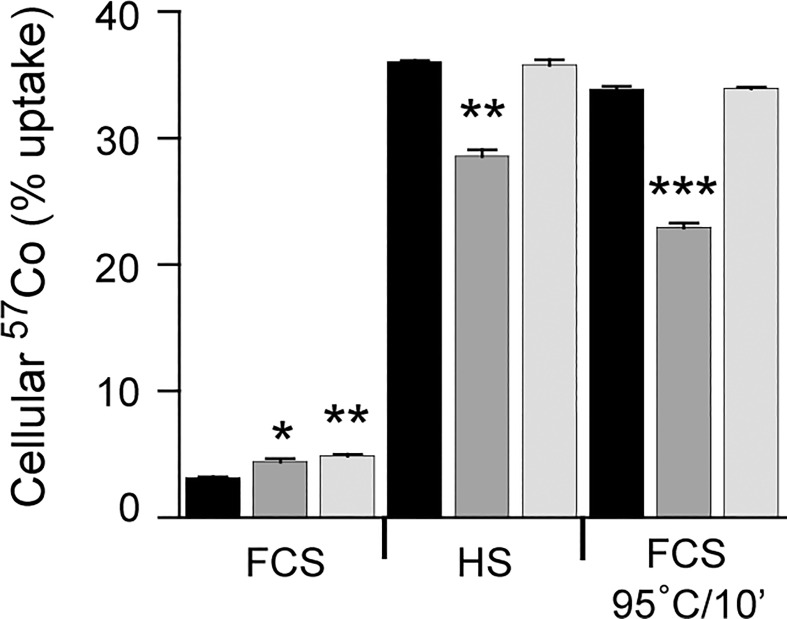
Assessment of HT1080 cellular ^57^Co-Cbl uptake in the presence of anti-transcobalamin antibody or anti-haptocorrin antibody. HT1080 cells were cultured in DMEM containing either 10% FCS, 10% heat-treated (95°C for 10 min) FCS, or 10% HS, for 48 h in either the absence of antibody (black bars) or the presence of anti-transcobalamin antibody (dark grey bars) or the presence of anti-haptocorrin antibody (light grey bars). The cells were harvested for ^57^Co analysis after 48 h. Data are mean values + SE (n = 3). * p < 0.05, ** p < 0.01, *** p < 0.0001.

In view of the previously described high binding affinity of HC for Cbl, these data suggest overall that the high levels of HC present in FCS competitively inhibits ^57^Co-Cbl binding to cell-derived TC. The heat treatment of FCS presumably denatures the structure of endogenous HC and other high-affinity Cbl-binding proteins (i.e. TC), thereby allowing ^57^Co-Cbl (that is either unbound or bound with low-affinity to other plasma proteins such as albumin [32]) to bind cell-derived TC. Our data also suggests that the HS content of endogenous TC contributes very little to the uptake of ^57^Co-Cbl over the duration of these experiments. To assess a contribution of cell derived TC in cellular ^57^Co-Cbl uptake, we added anti-TC antibodies (1/100 dilution) directly to the cell cultures and assessed cellular ^57^Co-Cbl levels after 48 h. Addition of TC antibodies inhibited cellular ^57^Co-Cbl uptake when HT1080 cells were grown in culture medium containing 10% HS or 10% heated FCS; however, this antibody mediated inhibition was not observed when HT1080 cells were grown in culture medium containing 10% FCS (Fig 6). Under the same experimental conditions we similarly added anti-HC antibodies (1/100 dilution) to the cell cultures and assessed cellular ^57^Co-Cbl levels after 48 h. Addition of HC antibodies did not inhibit cellular ^57^Co-Cbl uptake when HT1080 cells were grown in culture medium containing 10% FCS, 10% HS or 10% heated FCS (Fig 6). Addition of HC antibodies was associated with a small increase in cellular ^57^Co-Cbl levels (from 3.1% to 4.9%) when HT1080 cells were grown in culture medium containing 10% FCS (Fig 6).

Overall this suggests that under our experimental conditions, cell-derived TC is the key determinant of cellular ^57^Co-Cbl uptake. Based on this concept, we predicted that in the presence of DMEM only (i.e. lacking any form of FCS or HS), cellular ^57^Co-Cbl uptake should follow a similar time course profile to the serum-containing culture conditions, with cellular uptake coinciding with the appearance of TC in the cell culture supernatants. Preliminary evidence for this was also noted in the DMEM-only condition of the experiments described in Fi 2D, where in the absence of serum, significant ^57^Co-Cbl uptake was clearly detected at the 48 h time point. To assess this possibility, we conducted a time-course experiment in which we assessed both cellular ^57^Co-Cbl uptake and the proportion of ^57^Co-Cbl present in the > 30 kDa fraction of cell culture supernatants. We also used three different initial cell seeding densities: 10000 cells / well, 100000 cells / well and 400000 cells / well, that we predicted would yield a range of conditions that would compensate for potential reductions in cell growth due to nutrient deficiency afforded by the lack of serum in the medium (noting that in our routine experiments a seeding density of ~ 50000 HT1080 cells / well was used—see Materials and Methods for further details).

**Fig 7:**
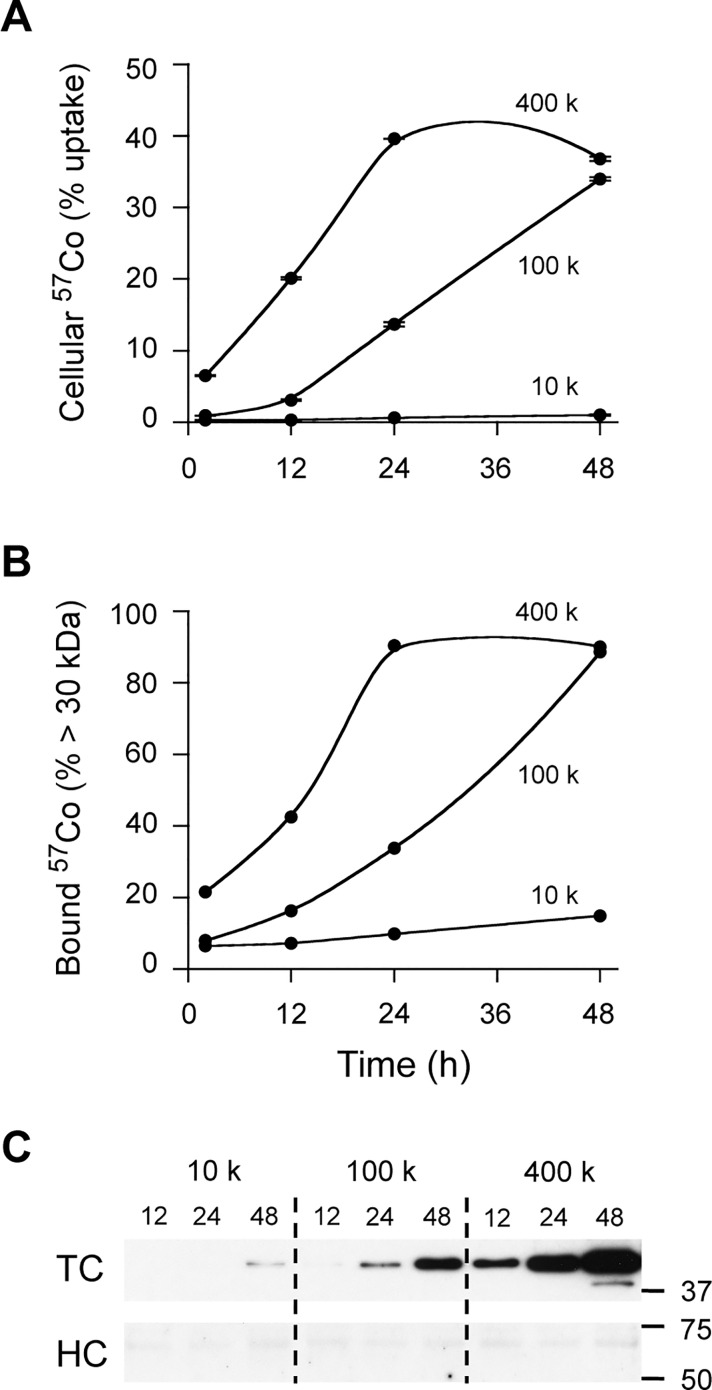
HT1080 cellular uptake of ^57^Co-Cbl in the absence of serum. HT1080 cells were grown at seeding densities of 1 x 10^4^, 1 x 10^5^ and 4 x 10^5^ cells per well. Serum-free DMEM growth medium was supplemented with ^57^Co-Cbl that was then incubated with HT1080 cells for 2 h, 12 h, 24 h and 48 h. ***A***, At the time points shown, cells were harvested and assessed for radioactivity. ***B***, The corresponding cell culture supernatants were also collected and were then centrifuged with a centrifugal filtration device and the proportion of radioactivity bound to proteins > 30 kDa assessed. The cell culture medium supernatants were also analysed for haptocorrin (HC) and transcobalamin (TC) western blotting. Positions of molecular weight markers (kDa) are given on the right side of the blots.

The results from these experiments show that HT1080 cell culture in DMEM alone is permissive for cellular ^57^Co-Cbl uptake (Fig 7A), and that the extent of uptake correlates well with both the amount of ^57^Co-Cbl present in the > 30 kDa fraction of cell culture supernatants (Fig 7B), and with the level of TC detected in the medium by western blotting (Fig 7C). In contrast, HC was either not detected (or detected as a weak signal close to the detection limits for this western blotting assay) in the cell culture supernatants (Fig 7C). Possible trace amounts of HC in the medium that were detected as weak signals on the blots did not correlate with incubation time, cell density or ^57^Co-Cbl uptake. This may therefore reflect residual HC remaining in the wells subsequent to the initial growth of the cells in the presence of FCS (that contains very high levels of HC as shown in Fig 5B). Additional studies with earlier time points were also conducted to probe for a possible biphasic kinetic for HT1080 cellular ^57^Co-Cbl uptake (i.e. to assess if cellular ^57^Co-Cbl uptake from DMEM followed a similar kinetic to the HS and heated FCS conditions shown in Fig 3B and 3C). When HT1080 cells were cultured in DMEM alone, the initial phase of cellular ^57^Co-Cbl uptake appeared to be slower than in either HS or heated FCS (S2 Fig), although the overall levels of ^57^Co-Cbl taken up were similar in the DMEM and serum-containing medium conditions (S2 Fig).

**Fig 8:**
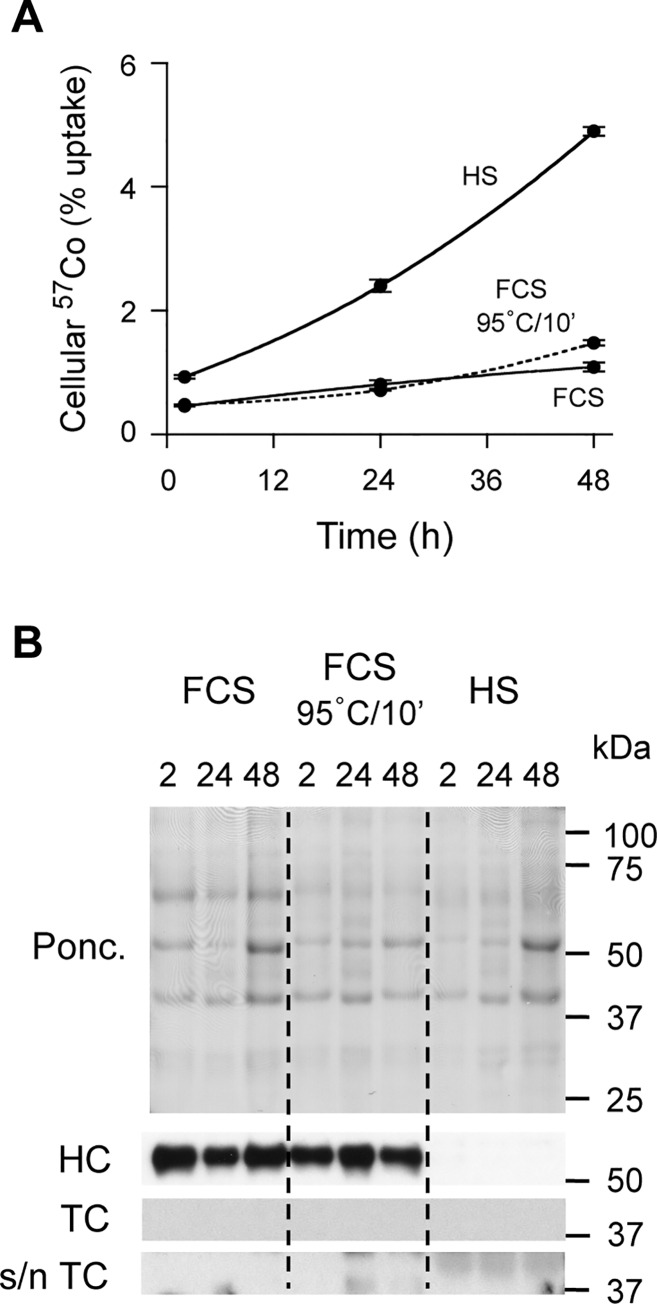
Cellular ^57^Co-Cbl uptake and haptocorrin and transcobalamin expression in AG01518 cells grown in the presence of either HS or FCS or FCS with heat treatment. ***A*,** HS, FCS or FCS heated at 95°C for 10 min before ^57^Co-Cbl addition, was used to assess cellular ^57^Co-Cbl uptake. Samples were assessed at 2 h, 24 h and 48 h. Data are mean values + SE (n = 3). ***B***, AG01518 cells were cultured as in “A” and assessed at 2 h, 24 h and 48 h for haptocorrin (HC), transcobalamin (TC) and cell culture supernatant transcobalamin (s/n TC). Positions of molecular weight markers (kDa) are given on the right side of the blots. Ponc., Ponceau red stained membrane.

In previous detailed studies, 34 different cancer cell lines were all demonstrated to express TC (and TCblR) in human tumor xenografts [33]. It is therefore possible that a wide variety of cancer-derived cell lines that produce TC may display similar characteristics to the HT1080 cell line regarding ^57^Co-Cbl uptake from growth medium containing FCS versus heated FCS. It is also possible that primary cell lines that are not derived from tumors may exhibit different phenotypes with respect to Cbl uptake and metabolism when cultured in HS, FCS or heated FCS. Although it is beyond the scope of the present study to screen a wide range of cell types, we have identified the human AG01518 fibroblast cell line (derived from neonatal human foreskin) as a relatively slow-growing cell line that does not appear to produce significant amounts of TC as determined by western blotting (Fig 8). In contrast to our studies with HT1080 fibroblasts, only 4.9% of ^57^Co-Cbl added was taken up by AG01518 cells grown in HS for 48 h (Fig 8A). This is approximately 86% less ^57^Co-Cbl uptake than in HT1080 cells (compare Fig 8A with Fig 3C). In addition, the amount of ^57^Co-Cbl taken up by AG01518 cells grown in FCS was only 1.1% over 48 h and this was only slightly increased to 1.5% when heated FCS (95°C/10 min) was used.

In agreement with the lack of TC in AG01518 cell lysates, we could not detect significant TC levels in the culture medium (Fig 8B). A trace of TC appeared to be present in the HS medium conditions, but this did not change over the 48 h time course and may therefore be due to the low levels of TC that are present in the initial HS growth medium samples. We also found that growing AG01518 cells in DMEM without any serum resulted in a level of ^57^Co-Cbl uptake that was similar to the FCS conditions (i.e. 1.4%, S2 Fig). This is also in contrast to the conditions where HT1080 cells were grown in DMEM alone (compare Fig 2A with Fig 2B). Overall, this suggests that in specific cell-types that do not produce significant amounts of TC, that HS is the preferable medium supplement to support ^57^Co-Cbl uptake, and that heating FCS makes very little difference to the overall cellular level of ^57^Co-Cbl.

## Discussion

Tracking cellular ^57^Co-Cbl uptake is a well established method for studying Cbl homeostasis, providing valuable information on both the regulation of Cbl endocytosis and the intracellular transport pathways. Consistent with previous studies, our current data reveal major differences in Cbl uptake depending on the serum type used in the growth medium. We extend earlier observations by showing that FCS actually inhibits HT1080 cellular ^57^Co-Cbl uptake, and that heat treatment ameliorates this inhibitory activity in experiments using HT1080 cells, a cancer-derived cell line that secretes TC. In cells that do not produce significant amounts of TC (e.g. as exemplified by the AG01518 fibroblast cell line used herein), FCS heat treatment does not appear to enhance ^57^Co-Cbl uptake. Our data are consistent with the idea that the very high level of HC in bovine serum is responsible for the inhibition of cellular ^57^Co-Cbl uptake. We suggest that bovine HC competes with cell-derived TC for ^57^Co-Cbl binding, and that since only a fraction of HC is not sialylated [34], and the asialoglycoprotein receptor is restricted to specific cell types [3], cellular ^57^Co-Cbl uptake is minimal in the presence of large amounts of bovine HC routinely used under *in vitro* cell culture conditions.

It is not clear why bovine serum has such a high concentration of HC as compared to HS. We speculate that since cattle are ruminant species and their gut bacteria are known to generate large amounts of Cbl and other Cbl analogues and corrinoid species [3, 35], a relatively large amount of HC is required to bind these molecules once absorbed into the blood stream and thereafter regulate their tissue storage and/or secretion.

We provide evidence that cell-derived TC is the major factor regulating cellular ^57^Co-Cbl uptake by HT1080 cells. This finding is in line with previous observations that many cell types have the capacity to secrete TC and that this correlates with the rate of cellular proliferation [3, 36, 37]. This is consistent with our observations in the present study where HT1080 cells secrete significant amounts of TC and have a reported doubling time of 20 to 24 h [26], whereas AG01518 cells did not secrete TC at levels that are detectable by western blotting and they have a reported doubling time of 3 to 4 days [27]. Our data show that even in the absence of serum, ^57^Co-Cbl uptake is maintained as long as sufficient TC is secreted by the cultured cells. Our data indicate a biphasic kinetic for cellular ^57^Co-Cbl uptake, the second phase (~ 12 h to 48 h) of which is highly correlated with the amount of TC detected in the HT1080 cell culture supernatants. An initial rapid rate of HT1080 cellular ^57^Co-Cbl uptake occurs in both the HS and heated FCS conditions (2 h to 8 h) that plateaus at ~ 12 h. Since the TC levels in FCS appear to be very low (Fig 5), it appears that serum TC might not drive this initial phase of cellular ^57^Co-Cbl uptake. In agreement with this, heat treatment of HS (which is predicted to denature both HC and TC) did not reduce this initial phase of HT1080 cellular ^57^Co-Cbl uptake (Fig 3). It is therefore possible that a small amount of this initial cellular ^57^Co-Cbl uptake could be independent of TC. Consistent with this, earlier work suggested fibroblasts could take up a low molecular weight form of ^57^Co-Cbl via a mechanism that was sustained for either 60 min [22] or 4 h to 8 h *in vitro* [24], depending on the experimental protocols employed. The rate for this initial phase of cellular ^57^Co-Cbl uptake was reduced in our current studies when HT1080 cells were grown in DMEM alone as compared to HT1080 cells grown in HS (S2 Fig). It may be that less specific pathways (e.g. pinocytosis) contribute to a small amount of uptake and this could possibly contribute to the cellular ^57^Co-Cbl levels that are detected (albeit to only ~5% of added ^57^Co-Cbl) when HT1080 cells were grown in FCS and such a pathway could also contribute to the low levels of ^57^Co-Cbl uptake by AG01518 cells.

It should also be noted that previous studies have shown that TC-specific uptake of ^57^Co-Cbl may proceed in the presence of FCS and the amount of cellular ^57^Co-Cbl detected after 48 h was found to be similar to what we have observed in the present study. For example, after 48 h, bovine aortic endothelial cells were shown to take up 0.59 fmol ^57^Co-Cbl / mg cell protein from a total (cells plus medium) of 8.89 fmol ^57^Co-Cbl / mg cell protein (i.e. 6.6% (see Table 1 of [38]). In this study, the initial cell growth medium was depleted of B12, and this may have enhanced the uptake of ^57^Co-Cbl. It is also clear that cells may convert endocytosed ^57^Co-CNCbl to different forms of Cbl (including MeCbl and AdoCbl), a proportion of which may be secreted back into the cell culture medium [38–40]. This would be predicted to have an impact on the rate and mechanism of ^57^Co-Cbl uptake through extended time-course experiments, and may be cell-type specific.

From a laboratory protocols perspective, even though a small amount of cellular ^57^Co-Cbl uptake is reproducibly detected when cells are grown in FCS, for studies that require isolation of intracellular organelles and Cbl transport proteins, increased labelling afforded by heat treated FCS has distinct advantages. This method of heat treating FCS (95°C/10 min) also has clear cost advantages over the use of HS or purified TC, in addition to the fact that a much greater proportion of the ^57^Co-Cbl required for these studies is actually utilised rather than being discarded in the spent culture medium. We also used adult bovine serum as a comparator for FCS in the experiments using HT1080 cells and found essentially identical results (i.e. like FCS, adult bovine serum was non-permissive for cellular ^57^Co-Cbl uptake but became permissive with heat treatment at 95°C/10 min, Zhao and Garner unpublished data).

Whilst our studies have utilised fibroblast cell lines, it is likely that the results will be relevant to other cell culture models as previous studies have shown FCS is not generally thought to be permissive for ^57^Co-Cbl uptake [21–25]. There may be certain exceptions where FCS is permissive for cellular ^57^Co-Cbl uptake. When studying cell types that express high levels of the asialoglycoprotein receptor (e.g. hepatocytes) that could potentially endocytose ^57^Co-Cbl bound to native HC present in FCS [41]. In addition, some cancer cell lines may produce HC [42] that may impact on the kinetics of cellular ^57^Co-Cbl uptake, although this was clearly not the case in the current study that used the fibrosarcoma-derived HT1080 fibroblasts that did not produce HC (Fig 7).

It is also possible that a proportion of cellular ^57^Co-Cbl uptake examined in the present work remains independent of the TC / TCblR pathway via mechanisms that remain to be defined. Related to this, genetic deletion of the TCblR in mice leads to a severe Cbl depletion only in the CNS [43], implying that other pathways may exist for cellular Cbl uptake in peripheral tissues. Whether such postulated alternate pathways are upregulated in response to TCblR loss in the abovementioned mouse studies or whether they may play a physiological role in humans remains to be defined. A soluble form of the TCblR (sCD320) has been detected in human serum at high pM concentrations [44, 45]. It is currently unknown if FCS and HS contain different levels of sCD320 and the extent to which this could impact on cellular ^57^Co-Cbl uptake under specific conditions remains to be defined.

Another aspect of cellular Cbl metabolism that has not been assessed in the present study pertains to the impact that the variable serum preparations may have on long-term cell growth and survival. Previous studies have shown that the addition of Cbl back to growth medium that is initially devoid of both Cbl and folate, results in an enhanced rate of cellular proliferation [46–48]. In these studies the enhanced rate of proliferation was generally in the order of an approximately 10% increase in cell number [46]. Other studies have shown that the amount of Cbl added to achieve enhanced cellular proliferation can be reduced 100- to 1000-fold if exogenous TC is also provided [49]. The data suggest that the amount of TC / TCblR expressed by individual cell types will play a role in determining Cbl uptake and Cbl-mediated enhancement of cellular proliferation. It is noteworthy, that a large number of cancer cell lines have been shown to express TC and TCblR in human tumour xenografts [33, 50], and it is possible that such cells could produce sufficient quantities of TC to compete with HC in FCS under *in vitro* conditions and in such cases heat treatment of FCS may not dramatically increase cellular Cbl uptake. Indeed, monoclonal antibody based interference of cancer cell up take of the TC-Cbl complex has been investigated as an antitumor therapy [49]. Clearly, it is advisable to assess Cbl uptake kinetics in planned *in vitro* studies that focus on intracellular ^57^Co-Cbl trafficking and metabolism.

## Conclusions

In conclusion, the present study reveals that FCS inhibits cellular ^57^Co-Cbl uptake *in vitro* and that this is most likely due to the high HC content of bovine serum. Furthermore, we show that heating FCS provides an easy method to improve cellular Cbl uptake, and that this is a cell-type dependent phenomenon. In the case of the main cell type studied here, HT1080 fibroblasts, cell-derived TC appears to be a major determinant of ^57^Co-Cbl uptake.

## Supporting Information

S1 Fig HT1080 cellular uptake of ^57^Co-Cbl in the presence of either FCS or HS with or without heat inactivation (56°C for 30 min). FCS or HS was either not heated or heated at 56°C for 30 min before ^57^Co-Cbl addition, then incubated with HT1080 cells for 48 h and compared to standard FCS and HS culture conditions. Data are from a single experiment.
(TIF)

S2 Fig Comparison of cellular uptake of ^57^Co-Cbl in the absence of serum or in the presence of HS. ***A***, HT1080 cells were incubated at 37°C with ^57^Co-Cbl in DMEM or DMEM containing 10% HS. ***B***, AG01518 cells were incubated at 37°C with ^57^Co-Cbl in DMEM or DMEM containing 10% HS. At the indicated times, the cells were harvested for ^57^Co analysis. Data are mean values + SE (n = 3).
(TIF)

## Abbreviations

AdoCbl: adenosyl Cbl
Cbl: cobalamin
DMEM: Dulbecco's modified eagle medium
FCS: fetal calf serum
HC: haptocorrin (also previously abbreviated as TCI, TCIII and R-binders)
Hcy: homocysteine
HS: human serum
IF: intrinsic factor
MeCbl: methyl Cbl
Met: methionine
5-methyl-THF: 5-methyltetrahydrofolate
Mm-CoA: methylmalonyl-coenzyme A
MMCM: Mm-CoA mutase
MS: methionine synthase
MRP1/ABCC1: multidrug resistance protein 1
PBS: phosphate buffered saline
SAM: S-adenosylmethionine
Succ-CoA: succinyl-coenzyme A
TC: transcobalamin (also previously abbreviated as TCII)
TCblR: CD320, transcobalamin receptor
THF: tetrahydrofolate
