# Supplementary figures and images for: Cell Type-Specific Modulation of Cobalamin Uptake by Bovine Serum
Source: PLoS One. 2016 Nov 28;11(11):e0167044. doi: 10.1371/journal.pone.0167044 (PMC5125665; doi:10.1371/journal.pone.0167044)

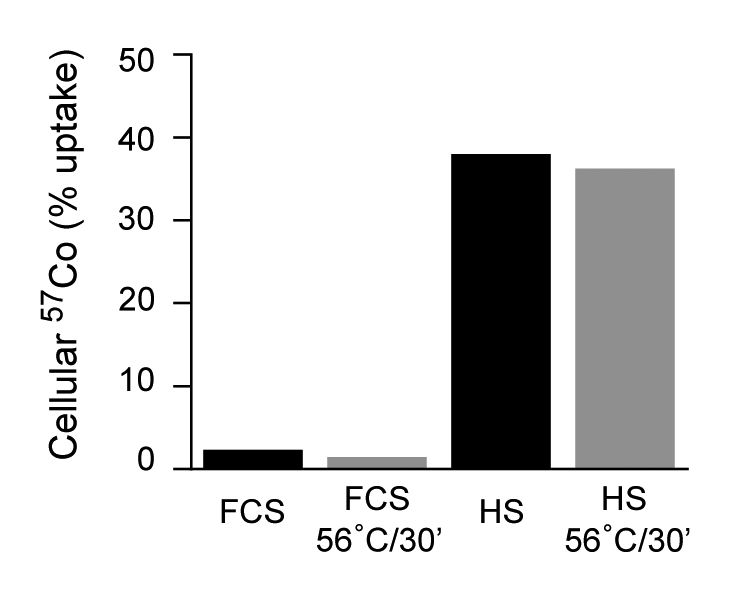

Supplement: S1 Fig — FCS or HS was either not heated or heated at 56°C for 30 min before 57Co-Cbl addition, then incubated with HT1080 cells for 48 h and compared to standard FCS and HS culture conditions. Data are from a single experiment. (TIF) [file pone.0167044.s001.tif]

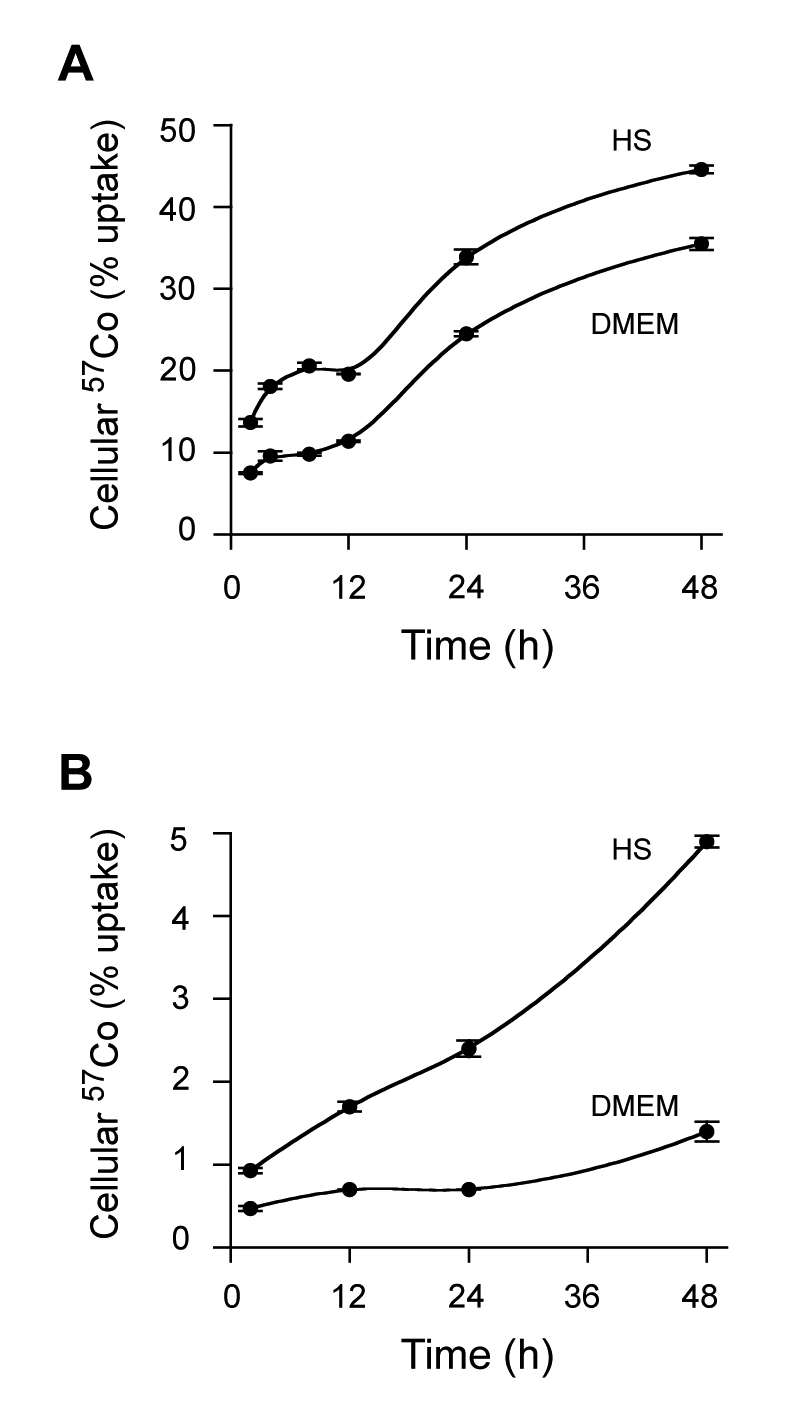

Supplement: S2 Fig — A, HT1080 cells were incubated at 37°C with 57Co-Cbl in DMEM or DMEM containing 10% HS. B, AG01518 cells were incubated at 37°C with 57Co-Cbl in DMEM or DMEM containing 10% HS. At the indicated times, the cells were harvested for 57Co analysis. Data are mean values + SE (n = 3). (TIF) [file pone.0167044.s002.tif]
